# St. Louis Medical and Surgical Journal

**Published:** 1845-09

**Authors:** 


### ST. LOUIS MEDICAL AND SURGICAL JOURNAL.

The August number of this Journal comes to us with an additional name upon the title page. Originally, it was conducted by Dr. M. L. Linton, who afterward was assisted by Dr. McPheeters, and now Dr. V. J. Fourgeaud is associated with them. The Journal has always been a respectable one, and, from the accession of Drs. McPheeters and Fourgeaud to its editorial management, will in future present still greater claims to patronage.

